# The effects of art design courses in higher vocational colleges based on C-STEAM

**DOI:** 10.3389/fpsyg.2022.995113

**Published:** 2022-11-11

**Authors:** Chen Qian, Jian-Hong Ye, Yi-Sang Lee

**Affiliations:** ^1^Dhurakij Pundit University, Bangkok, Thailand; ^2^Jinhua Polytechnic, Jinhua, China; ^3^Faculty of Education, Beijing Normal University, Beijing, China; ^4^Department of Industrial Education, National Taiwan Normal University, Taipei, Taiwan

**Keywords:** local culture, traditional Chinese culture, learning engagement, creative performance, creative self-efficacy

## Abstract

C-STEAM education is aimed at preserving local culture, while also improving students’ interests and skills in science, technology, engineering, art, and mathematics-related fields. Other goals are to cultivate students to solve complex and practical problems through interdisciplinary thinking or integrate learning subjects with local senses in the context. In the present curriculum implemented in China, STEAM education mainly focuses on K-12 education and kindergarten education, and it is not widely implemented in colleges and universities, and most of the existing courses are carried out in general technical courses such as robotics and 3D printing, and less in design courses, since the concept of STEAM education has just begun to be advocated recently. Nevertheless, STEAM courses are still limited to special educational systems and disciplines, even though these courses have been vigorously promoted in China. Thus, this study designed an innovative higher vocational college curriculum based on the interdisciplinary principle of C-STEAM, using art design as a meta-theme framework and integrating Chinese local culture. A single-subject quasi-experimental design method was used. A total of 45 students majoring in art design in a higher vocational college were invited to participate in this study. The teaching experiment lasted for 9 weeks. Through teachers’ teaching and, demonstration and students’ independent learning of C-STEAM knowledge in the field of art and design, the concept of C-STEAM was introduced to the creation of packaging design. At the same time, a model composed of six hypotheses was constructed, using the creative self-efficacy scale, learning engagement scale, and creative performance assessment as measurement methods, to discuss students’ participation in the art and design courses of higher vocational colleges based on the concept of C-STEAM integration of creative self-efficacy, learning engagement, and creative performance over time. The results showed that students with higher creative self-efficacy had higher learning engagement (cognitive, affective, and behavioral), and students with higher learning engagement performed better in terms of creative performance. The results of this study can help researchers and educators to focus on C-STEAM courses and provide suggestions for the cultivation of art and design professionals in higher vocational colleges.

## Introduction

With the advent of the era of the knowledge economy, the cultivation of human resources with creative expression ability has become the main theme of the cultivation of art and design talents in the 21st century (Shin and Jang, 2017; Shen et al., 2021). Learners are required to adapt an increasing amount of interdisciplinary knowledge and skills while working to generate new ideas, solve new problems, and create new products (Oldham and Cummings, 1996; He et al., 2019). However, the art design majors of some higher vocational colleges in China are mostly subject-centered, paying more attention to the learning of subject knowledge, without acknowledging students’ learning experiences. Thus, theory is not applied to actual practice, and students in higher vocational colleges cannot apply the knowledge they have learned to solve practical problems. “Knowledge” thus becomes “useless knowledge” (Linn and Hsi, 2000; Jia et al., 2021). A course that integrates scientific training and practical development not only helps to promote the creative expression ability of students in higher vocational colleges (Hong et al., 2016; Henderika, 2021), but also makes corresponding contributions to the cultivation of art and design professionals (Madden et al., 2013).

Science, technology, engineering, art, and mathematics (STEAM) education integrates art into the original STEM education, as art design has been shown by many scholars to play an important role in catalyzing STEM. It can promote students to understand from more and broader perspectives which can link across disciplines to foster creative expression, innovative thinking, and problem-solving (Radziwill et al., 2015; Root-Bernstein, 2015). Interpersonal skills such as teamwork are essential for social and professional development, cooperative communication, and adaptive capacity (Colucci-Gray et al., 2017; Michelle et al., 2022).

In recent years, STEAM education combining courses and thematic unit practice has become the mainstream teaching method (Workosky and Willard, 2015). In the development of STEAM courses in the learning environment in China, there is a phenomenon of excessive imitation of European and American courses. There is a lack of appropriate Chinese localization, poor inclusiveness of courses, no close integration with the existing curriculum system, and an inability to form acceptable courses combining issues such as sustainable development in China (Huo et al., 2020; Zhan et al., 2022). For this reason, Zhan et al. (2022) have proposed that the integration of disciplines education with Chinese local culture (Culture) as the goal should be combined with China’s national conditions. This approach is named C-STEAM and is a form of STEAM education with local cultural characteristics (Fan and Ye, 2022; Zhan et al., 2022). It is said that C-STEAM can effectively improve creative performance (Huo et al., 2020). However, relevant research lacks specific strategies, and the ambiguity of C-STEAM education, art, and design education and the cultivation of creative expression ability easily hinder the development of teaching, which means that practitioners cannot implement effective methods in C-STEAM education. It also affects the development of teaching and the realization of the ultimate goal of cultivating art and design talents in higher vocational colleges for local cultural education (Liao, 2016; Quigley and Herro, 2016). In order for the C-STEAM pedagogy to be effectively applied and implemented in art and design classrooms, an effective method of developing creative expression through C-STEAM education must be established. Therefore, this study chooses a practical art design professional course “Packaging Design” as the experimental research topic to discuss the impact of the C-STEAM education mode on students’ learning effectiveness.

Self-efficacy refers to an individual’s judgment and belief in whether he or she can accomplish the set goals in a specific task or situation, and is emphasized as an important factor in predicting learning outcomes (Siu et al., 2007; Wang et al., 2022). Creative self-efficacy is an individual’s confidence and evaluation of their ability to propose innovative ideas or achieve new results (Li and Guo, 2018). Furthermore, learning is a cognitive, affective, and behavioral experience (Frijda, 1986; Schaufeli et al., 2002). For example, learning engagement may be a prerequisite for students to participate in C-STEAM learning and is an important factor affecting academic achievement (Astin, 1985). Therefore, testing students’ learning engagement is a core element in measuring course quality (Kuh, 2001). Research has pointed out that creative performance is an individual’s comprehensive ability to generate new ideas, and discover and create new things. It is a necessary quality for successful creative activities (Li and Guo, 2018; Zhang et al., 2022). Based on the above reasons, this study focuses on the aspects of students’ self-efficacy, student engagement, and creative performance to reflect learning effectiveness.

Based on the above, the purpose of this study is to apply a single-subject quasi-experimental design method to test the acquisition of interdisciplinary knowledge, explore the relationship between creative self-efficacy, learning engagement and creative performance, and then propose a research model to explain the learning effects of students after the experiment of art design courses in higher vocational colleges based on C-STEAM, so as to achieve a win-win result of cultural focus, discipline development, and the cultivation of art design talents in higher vocational colleges.

## Literature review

### C-STEAM & Shangshan culture and art design

C-STEAM emphasizes the internal integration of inter-disciplinary knowledge and thinking methods oriented towards the inheritance of local culture, rather than simply imposing traditional culture on existing STEAM courses (Huo et al., 2020). Chinese local culture is the spiritual blood of the Chinese nation. It embodies the values and ethics generally recognized by the local people. It embodies profound ideological connotations, artistic values, and aesthetic habits, as the sources for creative expression and the root of life (Huo et al., 2020; Zhao et al., 2022). “Wannian Shangshan” is a prehistoric cultural site with the most local characteristics, Chinese significance, and world influence. There are 20 sites in total, including 14 sites in Jinhua city, Zhejiang province (Qian and Wang, 2022). The Shangshan site group has excavated a variety of living utensils and production tools, such as pottery, stone tools, wooden buildings, rice remains, and rice ear base plates, which have obvious regional attributes (Jiang, 2007). C-STEAM education is based on science, recognized reality, integrations of multi-disciplinary knowledge, and the focuses on the multi-sensory experience that guides students to unite their hearts and hands. It seems to be in line with the characteristics of Shangshan culture and the cultural experience and diverse learning forms behind it. This creates the conditions and opportunities for nurturing and developing students’ creative expression (Watson and Watson, 2013; Huo et al., 2020).

The art design major designed by China’s higher vocational colleges has the advantages of local cultural heritage and innovation, and has an extraordinary influence on the cultivation of art and design talents with local characteristics. It is obliged to shoulder the major responsibility of cultural heritage and protection construction (Qian and Wang, 2022). Art design is a highly comprehensive activity that needs to solve many complicated problems. It involves the designer’s comprehensive qualities such as creative expression, imagination, and so on (Zeisel, 2006), and eliminates complicated problems requiring corresponding solving skills and innovation ability (Hong et al., 2019a,b). Technology and engineering in C-STEAM education are directly related to problem solving, optimization, and design (Hernandez et al., 2014; Sha et al., 2021). At the same time, art design is an art subject that requires a great deal of knowledge accumulation as the basis for learning. Taking packaging design as an example, the production process is rigorous and complex. It is necessary to consider packaging materials, production techniques, shape and structure, appearance decoration, size, printing, and so on, that all fit perfectly with C-STEAM. Therefore, this study discusses the connotation of C-STEAM in the art and design topic and constructs it into a whole learning curriculum activity, as well as the creative performance of students.

### Creative self-efficacy

Bandura (1977) was the first person to propose that self-efficacy is one of the key factors affecting the way individuals learn, and plays a pivotal and unique role in each task (Carberry et al., 2010; Fryer and Ainley, 2017). Creative self-efficacy is a concept put forward by Tierney and Farmer (2002) that integrates self-efficacy and creative performance (Beghetto, 2006). When faced with challenges or obstacles, individuals with high creative self-efficacy can generate strong internal motivation; on the contrary, people with low creative self-efficacy are more likely to avoid creative tasks or to give up. The idea is that the difficulties cannot be solved, and the bottleneck is difficult to break through (Tierney and Farmer, 2004; Beghetto, 2006). Therefore, this study uses creative self-efficacy to measure students’ belief in their ability to complete packaging design tasks.

### Student engagement

Astin (1985) first proposed the theory of learning engagement, which was further refined by Kuh (2001), which refers to the energy and time spent by students in activities with educational goals, and is the process of students’ thinking, emotion, and behavior in learning. Schaufeli et al. (2002) mentioned that learning engagement is a mental state manifested from within an individual during learning. Different learning engagement reflects the different learning vigor and active level of participants, mainly including emotional and behavioral components. The two parts are the quality of emotional experience and the performance of positive behaviors when learners start and participate in learning activities (Schaufeli et al., 2002; Jimerson et al., 2003). Emotional engagement involves the psychological feelings of learners when they participate in learning and is an expression of emotional responses and attitudes to learning, including enjoyment, interest, and satisfaction (Jimerson et al., 2003; Liu et al., 2021). Behavioral engagement is the persistent behavioral conditions exhibited by learners while facing difficulties or failures (Schaufeli et al., 2002; Kuh, 2009). Fredricks et al. (2004) further proposed that learning engagement includes not only emotion and behavior, but also cognitive orientation, which is related to the understanding of learning and is the degree of mental effort that learners generate to ensure that they achieve their learning goals. It has also been shown to modulate the learning process through cognitive strategies (Schaufeli et al., 2002). Therefore, this study used learning engagement to explore learners’ engagement in C-STEAM integration into a packaging design courses.

### Creativity performance

Originally proposed by Guilford (1950), creative performance is an individual’s potential psychological ability to generate and propose new ideas, new solutions, and other creative activities in a specific field or social and cultural context (Amabile, 1983; Shalley et al., 2004). At the same time, Lin (1999) further defined creative expression as the use of all known information for a specific purpose to produce thinking results with distinctive, unique, social, or personal value, which can be new products, new processes, or new ideas. Technology can also be new ideas, new concepts, or new theories (Zhao et al., 2021; Zhang et al., 2022). This study followed the definition of creative expression in Lin (1999), and examined the learning effect of the integration of C-STEAM into a packaging design courses.

## Materials and methods

### Course design

#### Course content

The syllabus of “Packaging Design” refers to the “14th Five-Year Plan” textbook “Packaging Design” for colleges and universities edited by Liu et al. (2022). It mainly involves the six subject areas of culture (C), science (S), technology (T), engineering (E), art (A), and mathematics (M) (Fan and Ye, 2020). Themes integrated into Shangshan culture, such as pottery art, wooden architecture, rice history, and so on, could be used as the background, themes, and results of the design activities. The students learned by doing and playing. It was ensured that students would establish interdisciplinary relationships, and connect and deepen their cultural experience (He and Wong, 2022; Qian and Wang, 2022). The main goal of this course was to guide students through the learning experience, from the perspective of art design, to cultivate their comprehensive design ability from packaging material selection, structure production, to appearance decoration, and to turn their thoughts into “personalized” works with long-term vitality. The whole process reflects the students’ ability to express their own thinking and emotional experience in the works in the form of materialization, not just simple production skills. In this study, a 9-week teaching experiment was conducted to convey the core connotations of C-STEAM education and Shangshan culture to art and design students, as shown in Table 1.

**Table 1:** Course flow chart.

Courses	Fields	Contents
The fundamental descriptions of art design of the packaging	C	The content of Shangshan spirits
	S	The material characteristics and history of the packaging
	T	The art skills and forms of the packaging
	E	The functions and structures of the packaging
	A	The style and expression of the packaging
	M	The size of the packaging
The design of visual delivery of the packaging	C	Shangshan Cultural Element Mining
	S	Regional packaging material selection
	T	Craftsmanship
	E	Internal and external structure of packaging
	A	Image and Graphics Rendering and Arrangement
	M	Size calculation
Packaging design strategy and process	C	Analysis of Shangshan Culture Elements
	S	Product-Oriented Material Selection
	T	Sketch computer design draft
	E	Packaging design and production process
	A	Highlight product branding graphics, words, and colors
	M	Market research and data summary
Series Shangshan product packaging creation	C	Refinement of Shangshan Cultural Elements
	S	Regional material selection and production
	T	Printing proofing
	E	Single and series gift box structure design
	A	Cultural element modeling performance
	M	The corresponding proportion of finished products

#### Curriculum planning

In the first week (eight class hours), a pre-test of the relationship between creative self-efficacy, learning engagement, and creative performance was conducted. A large number of domestic and international classical cases were brought into the teaching of the basic connotation of packaging design. The purpose and function of packaging, popular science pictures and texts were compiled by researchers who imported Shangshan culture. The integrated packaging design included several C-STEAM concepts. For instance, Shangshan spiritual connotation and natural aesthetics belonging to the culture; packaging material characteristics and history belonging to science; packaging technology and form belonging to skills; internal function and external structure design of the packaging belonging to engineering; packaging design style and expression belonging to art, and packaging specifications belonging to mathematics.

In the second to third weeks (16 class hours), students conducted independent learning of packaging visual communication design through the Smart Vocational Education network platform. Based on the recorded videos, students self-learned graphics, text, color, visual arrangement, and packaging materials and structures according to production procedures. The self-learned criteria also included practical exercises which linked C-STEAM with the professional field knowledge of packaging design, including cultural elements mining, regional packaging material selection, production technology, structural design, image and graphic drawing and arrangement, and size calculation. Then, based on homework and relevant students’ feedback, offline one-on-one tutoring was provided to students who had difficulties integrating C-STEAM with packaging design.

The fourth to fifth weeks (16 class hours) were focused on packaging design strategy and process learning. Students needed to know procedures including: (1). How to integrate C-STEAM and packaging design knowledge. (2). How to choose green concept-oriented packaging materials. (3). How to familiarize themselves with sketches and computer design drafts. (4). How to design draft revisions to optimize the production process to printing. (5). How to make packaging based on product characteristics, and how to master the graphics, font image and image color used to highlight the brand and understand the internal and external structure of packaging. Lastly, the branded products of the Shangshan ruins sites were determined as the target design projects with the geographical advantages while carrying out the market research. Either the direct or indirect research method was selected to do the target market research, commodity and packaging research, and product and commodity analysis. Eventually, a market research summary was composed.

**Figure 1:**
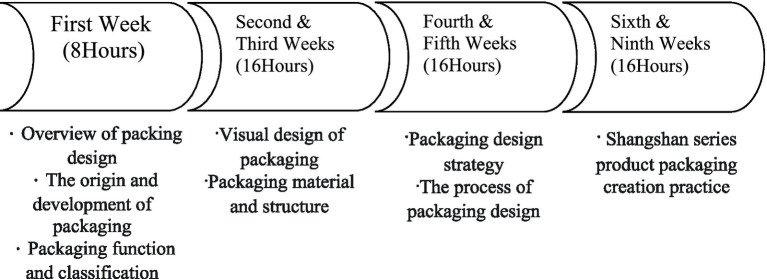
Curriculum map.

The sixth to the ninth weeks (32 class hours) were the series of product packaging creation practice and the completion of the post-test. First of all, the main issue which needed to be considered was how to customize the packaging design of the peripheral products or gift boxes related to a single Shangshan ruins sites group according to product functions and cultural characteristics. Second, the learning requirements needed to be clarified. In the first 5 weeks, practical exercises and market research were taught. Based on the previous lessons of the theoretical knowledge of packaging, Shangshan culture should be integrated and a series of no <5 pieces of packaging works should be created. Third, specific procedures for the requirements should be formulated. Time to design should be reasonably consumed. The elements of Shangshan culture that were learned from cultural innovation cases should be well considered, so the entry point of packaging design could be easily found. Fourth, hand-drawn drafts should be made instead of hand-made samples. Fifth, Photoshop, Illustrator, and other drawing software should be used for the computer design and production. The mouse or tablets should be used to trace and outline the draft on the computer step by step. Sixth, the design draft should be revised and optimized by carefully improving the packaging size to fit the corresponding proportion. Each detail should be handled until the final expanded drawing and three-dimensional rendering were completed (Figure 1). Seventh, students presented their final work, and shared the design ideas and the concepts of their work with others (Jia et al., 2021).

Specifically, the “Packaging Design” course consists of four course units including basic overview of packaging design, packaging visual communication design, packaging design strategy and process and series of product packaging creation. Each course unit tightly links with the integration of Shangshan culture with professional domain knowledge, material properties and material selection gist, production techniques and tool application professional domain knowledge, structure and modeling professional domain knowledge, artistic aesthetic professional domain knowledge, and packaging size professional domain knowledge that are all carried out by teaching and learning activities (as shown in Table 2).

**Table 2:** Lesson plan.

Course unit 4: The packaging design creation for Shangshan culture product line		
Refinement of Shangshan cultural elements (C)	A	The graphic symbolization of Shangshan Culture is to identify the cultural characteristics, extract and summarize the most representative and symbolic elements, and use them skillfully and vividly to form a visual impact
	B	The use of regional materials from Shangshan region highlights the original characteristics of the packaged products
	C	The characteristics of Shangshan culture are adopted in the form of miniature or bionic to enhance the creativity of the packaging shape
Material properties and material selection gist (S)	A	Use straw, leaves, bamboo strips and other packaging materials with local characteristics to make the products more authentic
	B	Natural materials and handmade materials are coordinated to highlight regional characteristics
	C	Single material and the composite material merge into each other to enhance the visual impact
Production techniques and tool application professional domain knowledge (T)	A	Draw structural sketches based on product dimensions
	B	Make samples by hand, adjust the size in time, and tag relevant data
	C	Draw the required graphics, make related images and create related texts in the structure expansion diagram
	D	Select drawing software, and use the mouse or tablet to trace and outline the draft step by step on the computer
	E	Proofing the finished product, perfecting the finished product size or the corresponding proportional relationship, handling the details of the packaging, and completing the final unfolded drawing and 3D effect drawing
Structure and modeling professional domain knowledge (E)	A	Designing the packaging structure in miniature is one of the traditional ways to reflect the local culture
	B	Additional accessories are hung or inlaid, etc. to make the container more diversified
	C	The cover of the structure is various to fully make the package fancier like the icing on the cake
Artistic aesthetic professional domain knowledge (A)	A	The visual arrangement method of the packaging layout is divided into the oriented method and the visual center method, focusing on the visual process in line with people
	B	The packaging layout forms include horizontal, vertical, split, free and inclined
	C	The layout design principles focus on unity, integrity, coherence and vividness
Packaging size professional domain knowledge (M)	A	The liquid packaging size is measured in milliliters. For conventional 500 ml liquid products, the general size is 150 mm in length, 100–120 mm in width, 300 mm in height, and has a “welding” of about 10 mm
	B	For medium and large packaging, the size of the inner box is X1-Xma*Nx + d (Nx-1) + k + T. X1 is the inner size of the carton, Xma is the size of a single content, D is the tolerance coefficient of the content, Nx is the number of the contents arranged in a certain direction in the carton, k is the correction factor of the inner size of the box, and T is the total thickness of the spacer or buffer (All lengths are in mm)
	C	The barcode should be a standard size of 37.29 mm * 26.26 mm, and the scaling ratio should be 0.8–2.0 times. The key point is that the height of the barcode symbol should not be arbitrarily truncated

#### Overview of packaging design

**Figure 2:**
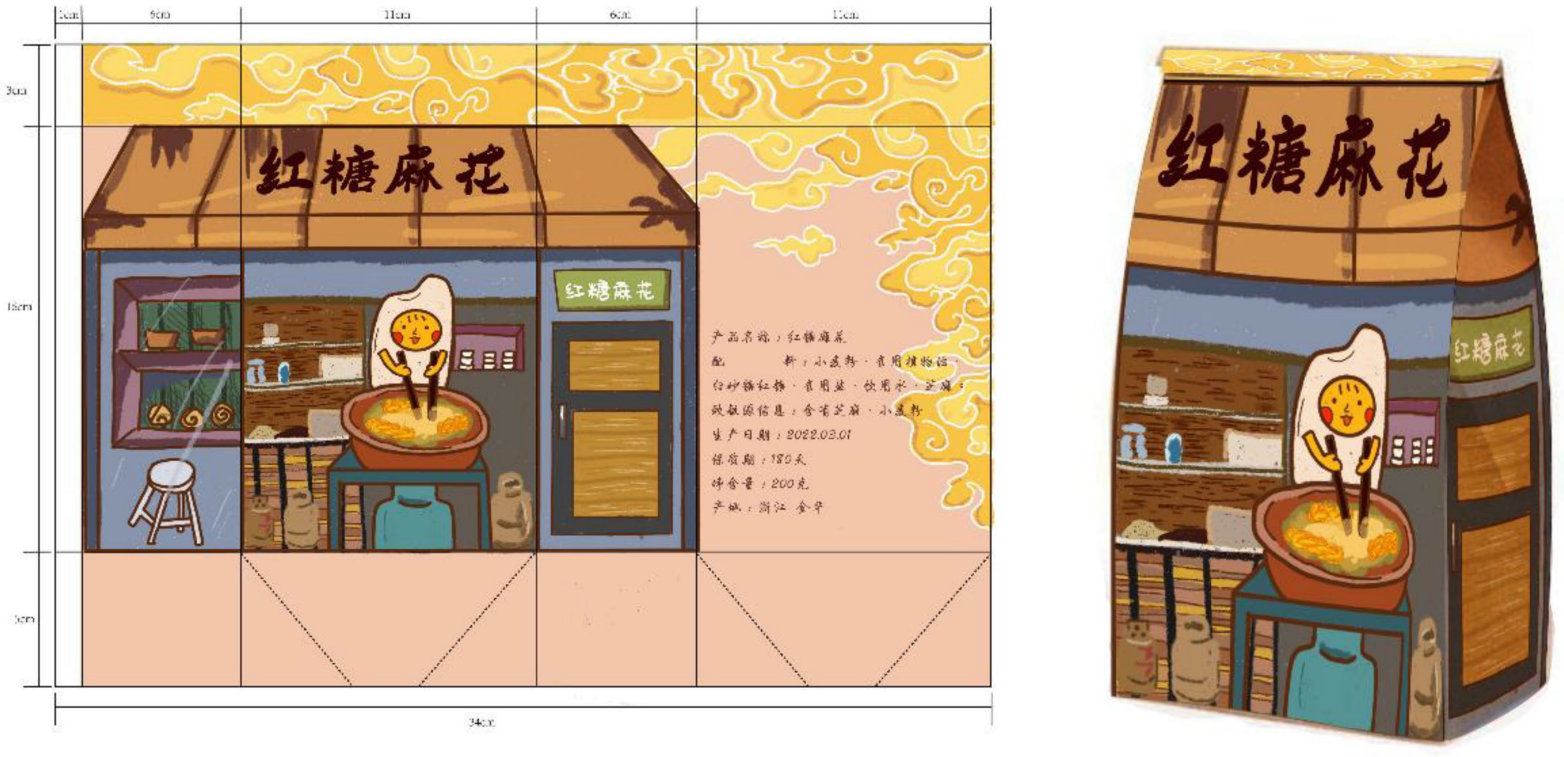
Laojiekou packaging design.

**Figure 3:**
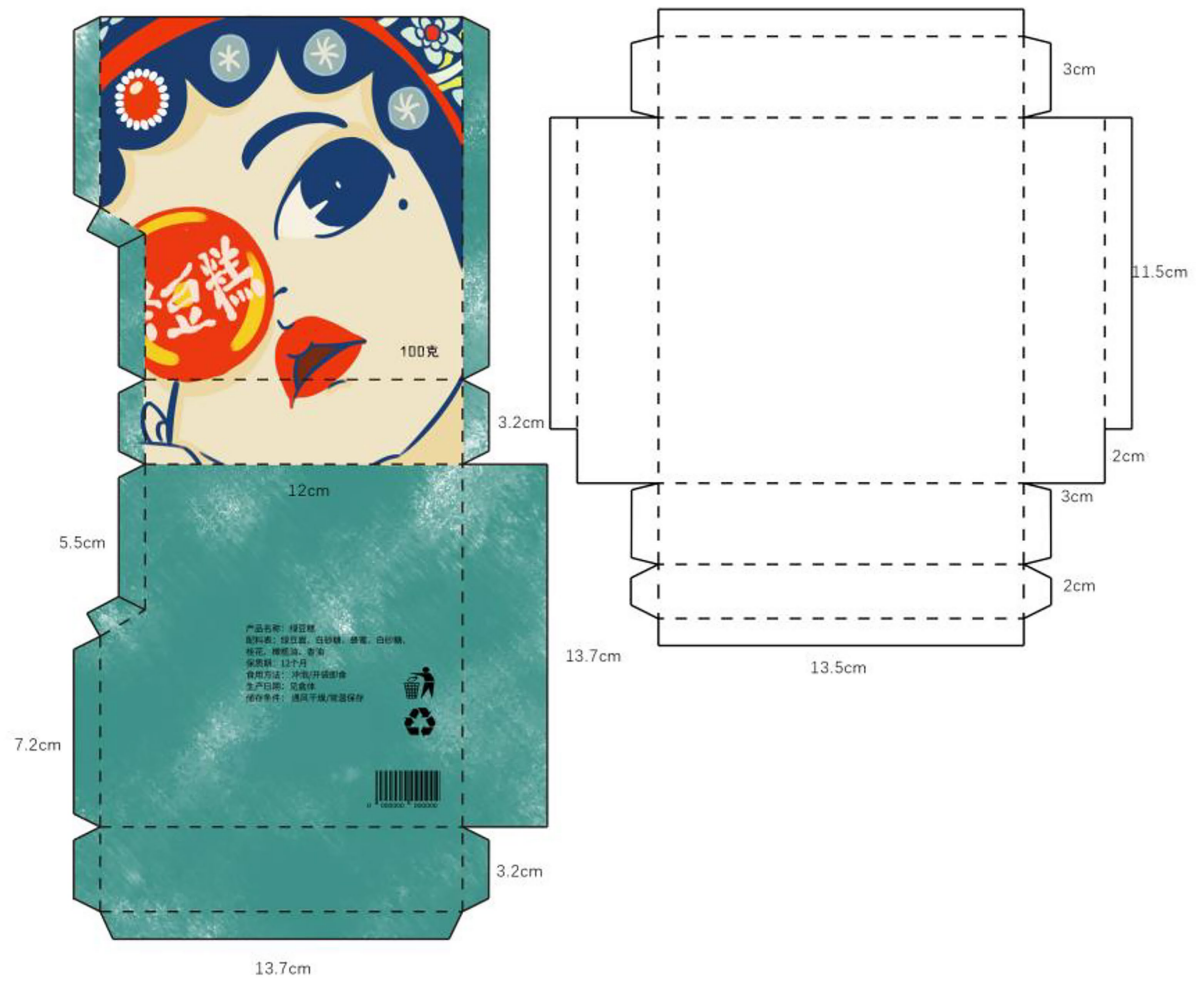
Mung bean cake packaging design.

**Figure 4:**
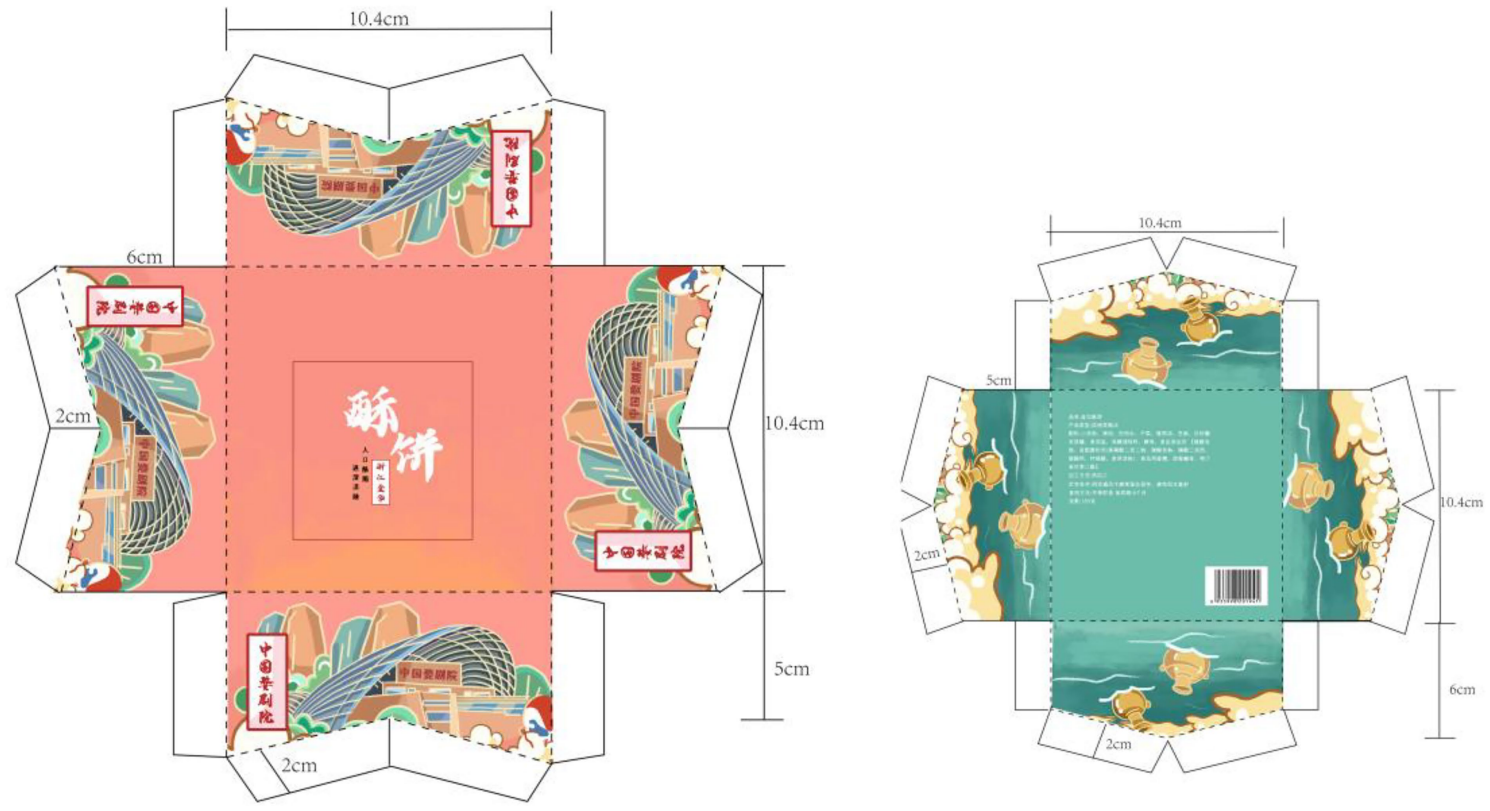
Shortbread packaging design.

**Figure 5:**
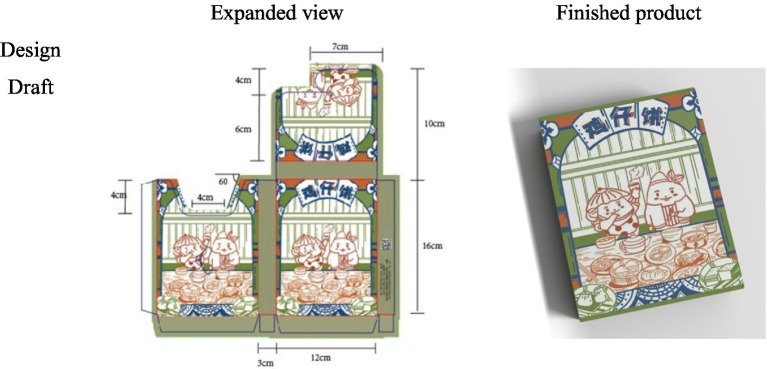
Chicken pie packaging design.

The final packaging design works completed by the students are shown in Figures 2–5. The C-STEAM integrated art design course concept was applied to the packaging design creation of local famous products in Shangshan. The six disciplines of culture, science, technology, engineering, art, and mathematics were integrated and presented. The most representative and symbolic Shangshan cultural elements were from either the extraction or induction. Packaging materials were selected according to the product characteristics. The packaging design and production process were strictly implemented with the consideration of the ergonomic perspective and product characteristics. Modeling design with the overall packaging style referred to arranging pictures, texts and colors, and mathematical concepts to measure actual proportions and calculate packaging dimensions and other tedious processes (Zhou and Zhou, 2019).

For example, the process of packaging design of Laojiekou twist production created in Figure 2 was drawn with the main image of “Shangshan Xiaobai.” The packaging and decoration design was combined with the wooden architectural scene of Shangshan. The bagging techniques learned during the course were used for application. As another example, Figure 3 represents the packaging design of mung bean cakes that skillfully combined traditional local culture and modern painting styles, especially in the use of color. The red color of pottery, the yellow color of rice ears and the green color of rice in the Shangshan Cultural Site were displayed in the packaging. Moreover, Figure 4 shows the packaging design of Jinhua Shortbread which referred to the observation and measurement of the existing buildings in Shangshan Archaeological Site Park and the houses unearthed from the archaeological sites. The packaging structure, pattern and color reflect the local characteristics of Shangshan. Figure 5 represents the packaging design of chicken pies. The characters are outlined with colored lines. The scenes were drawn with large colored blocks, which combined the culture of Shangshan with the characteristics of the food. All of these packaging appearances represent good visual effects.

### Research method and process

Due to the limitations of research ethics and budget, random assignment cannot usually be applied in the research of teaching practice, so a quasi-experimental design method was used to evaluate the experimental effect (Kim and Steiner, 2016). This study adopted the quasi-experimental design method of a single subject group, and the teaching experiment lasted for 9 weeks (a total of 72 class hours). The student questionnaire relative to creative performance, the teaching experiment, the progress of the participants’ creative performance, and the packaging design works were collected and analyzed as qualitative data according to the multi-assessment results to further verify the quantitative data analysis results.

### Research model

**Figure 6:**
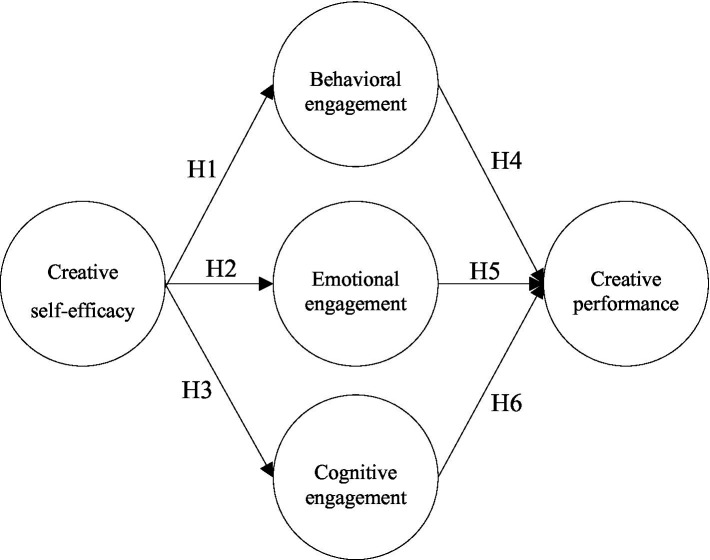
Research model.

The theory of learning engagement is regarded as a key factor in the learning process and provides an important framework model for related research content (Astin, 1985; Connell, 1990). Among them, self-efficacy, as a core element of the learning engagement theory, can trigger learning engagement. The stronger the individual’s beliefs, the greater the quantity and quality of their engagement. It is considered to be a highly efficient predictor of learning engagement (Tierney and Farmer, 2002). In addition, learning engagement can directly or indirectly affect educational outcomes, and individuals who devote more time to creative activities can demonstrate higher levels of creative performance (Beghetto, 2006; Grosser et al., 2017). Therefore, on the basis of relevant literature research and theoretical discussions, this study was based on the theory of learning engagement and the relevant literature on creative self-efficacy, learning engagement, and creative performance. Creative self-efficacy was determined by analyzing the three dimensions of learning engagement (cognitive engagement, emotional engagement, and behavioral engagement) and the five research variables of creative performance. A research model of students’ creative performance in Chinese higher vocational colleges is shown as Figure 6.

### Research hypotheses

#### The impact of creative self-efficacy on learning engagement

Self-efficacy has an important impact on learning engagement (Ouweneel et al., 2013; Gan and Jia, 2015). Self-efficacy focuses on self-learning and skill development and is the result of individuals’ self-assessment and evaluation of their own abilities. Nevertheless, evolution in turn negatively moderates people’s choices and levels of engagement in behaviors (Middleton et al., 2019; Asghar et al., 2022). Creative self-efficacy is an extension of self-efficacy in the field of creative performance (Beghetto, 2006). During the process of creation, creative self-efficacy is a very important inspirational factor. It prompts one’s recognition, emotion, and behaviors, so individuals can devote more time to creative activities (Beghetto, 2006; Grosser et al., 2017). Gan and Jia (2015) further pointed out that creative self-efficacy and learning engagement are significantly positively correlated. According to the above literature, this study added relevant variables and proposed the following hypotheses:

*H1:* Creative self-efficacy has a significant positive effect on cognitive engagement.

*H2:* Creative self-efficacy has a significant positive effect on emotional engagement.

*H3:* Creative self-efficacy has a significant positive effect on behavioral engagement.

#### The impact of learning engagement on creative performance

Learning engagement has a positive predictive effect on creativity (Gan and Jia, 2015). Relevant studies have confirmed that learning engagement is a good “prediction period” for creativity, which is not only conducive to stimulating the positive qualities of students’ creativity, but also promoting students’ comprehensive development (Schaufeli et al., 2002; Ouweneel et al., 2013). Creative expression is the behavioral expression of creative potential (Oldham and Cummings, 1996; Choi, 2004), which is to put one’s own ability and energy into novel and useful ideas (Choi, 2020). Wang and Eccles (2011) and Li and Liu (2014) further pointed out that with the comparison among students with different level of learning engagement, the more cognitive, emotional and behavioral students invest in creative activities with higher effort requirements, the greater impact on students’ creative performance. This study used creative performance to discuss the learning effectiveness of learners in the integration of C-STEAM into design courses, and analyzed the relationship between learning engagement and creative performance, and proposed the following hypotheses:

*H4:* Cognitive engagement has a significant positive impact on creative performance.

*H5:* Emotional engagement has a significant positive impact on creative performance.

*H6:* Behavioral engagement has a significant positive impact on creative performance.

### Participants

This study was carried out in the art design major of a vocational and technical college in Jinhua City, Zhejiang Province, China from February 21 to April 21, 2022. There were 45 s-year students, including 14 males (31.11%) and 31 females (68.89%), who participated in this “Packaging Design” study. The average age was 19.7 years (standard deviation 2.5 years). The reason for the uneven gender ratio in the study was that the majority of Chinese students majoring in art and design are women (Fan and Ye, 2020). Before this course was launched, none of the vocational art students had any learning experience related to C-STEAM education and Shangshan culture.

### Measurement

The study data were collected through a questionnaire survey, using the 5-point Likert scale design (1–5 means *strongly disagree* to *strongly agree*). All items were revised based on previous theories and research tools. A secondary content validity review was conducted by three art and literature experts with senior professional titles to determine the validity and legibility of the text. The content of the questionnaire was divided into three parts: the first part was the explanatory information of the questionnaire; the second part was the survey of the background information of the participants; and the third part was the survey of the feedback of the study participants, which was also the main part of the questionnaire.

#### Creative self-efficacy

The measure of creative self-efficacy was the scale modified from the one prepared by Hong et al. (2019a,b), with a total of eight items. The reliability of the questionnaire had a Cronbach’s *α* value >0.7 which indicated that the statistical data of the study had good reliability (Nunally and Bernstein, 1978). According to Hair et al. (2014), a construct validity value exceeding 0.7 represents the internal consistency reliability of the study. The analysis results of this study showed that the Cronbach’s *α* coefficient was 0.93 and the critical ratio (CR) was 0.94 (as shown in Table 2), which met the criteria. According to the standard proposed by Hair et al. (2014), when the internal validity was tested, the items with a factor loading (FL) less than 0.5 were excluded; five items were finally determined for this study. According to Fornell and Larcker (1981), when the reference values of average variance extracted (AVE) and FL are both greater than.6, the construct studied can be determined to have good convergent validity. In this study, the AVE was 0.75 and FL was 0.86 which fit the standards.

#### Learning engagement

Engagement in the learning process can be viewed as a multidimensional construct that includes the cognitive, emotional, and behavioral levels (Fredricks et al., 2004). The learning engagement scale of this study was modified from the competition engagement scale by Ye et al. (2020) to measure learners’ perceptions of three constructs of learning engagement in packaging design courses. All constructs of the scale need to be greater than 0.7 to indicate good reliability (Nunally and Bernstein, 1978). According to Anderson and Gerbing (1988), CR values greater than 0.6 indicate that the study has internal consistency reliability. The AVE value and construct validity of the three constructs of this study were: Cronbach’s *α* values between 0.8 and 0.94; and CR values between 0.91 and 0.96 (see Table 2).

Factor loading should be >0.5 to prove validity (Hair et al., 2014). In this study, the number of cognitive engagement items was reduced from eight to six; the number of emotional engagement items was reduced from nine to eight; and the number of behavioral engagement items was reduced from nine to eight. Hair et al. (2014) suggested that the AVE and FL values should both exceed 0.5 to have convergent validity. In this study, the AVE for cognitive engagement was 0.73; the AVE for emotional engagement was 0.73; the AVE for behavioral engagement was 0.64; the FL for cognitive engagement was 0.85; the FL for emotional engagement was 0.87; and the FL for behavioral engagement was 0.8. All of the values fit the criteria.

#### Creative expression

The creative expression construct of this study adopted the revised artistic creativity evaluation standard (Li and Guo, 2018) as the evaluation index of packaging design works. A total of five people in related fields, including three full-time art design teachers, one industry expert with local cultural research expertise, and one part-time teacher from industry were entrusted to evaluate the creative performance of the higher vocational college students and further verify the results of quantitative data analysis. The evaluation criteria included seven dimensions: novelty, suitability, technicality, imagination, aesthetics, emotion, and comprehensive impression. Each dimension had a maximum of five points and a minimum of one point. The total score of creative performance was the average score of the seven dimensions (Table 3).

**Table 3:** Reliability and validity analysis.

Construct	*M*	SD	*α*	CR	AVE	FL	*t*
	–	–	>0.70	>0.70	>0.50	>0.50	>3
Creative self-efficacy	3.96	0.61	0.93	0.94	0.75	0.86	5.42–10.24
Behavioral engagement	4.02	0.63	0.92	0.94	0.73	0.85	6.28–9.70
Emotional engagement	4.04	0.58	0.94	0.96	0.73	0.87	4.22–10.28
Cognitive engagement	4.04	0.55	0.88	0.91	0.64	0.80	5.12–8.75

## Results

Structural equation modeling (SEM) is the main analytical tool for measuring causal relationships among latent variables (Mueller and Hancock, 2018). Among many structural equation model data analysis methods, partial least squares structural equation modeling (PLS) is regarded as an analytical method particularly suitable for testing new theories (Streukens and Leroi-Werelds, 2016). Therefore, this study firstly used IBM SPSS 23.0 for correlation analysis and reliability and validity analysis, and then used VisualPLS 1.04b1 to analyze the structural equation model.

### Analysis of creative performance

According to the internal scoring of all students’ packaging design works by field experts, the statistical results show that the average score of creative performance of 45 vocational students was 3.54, with the highest score of 4.25 and the lowest score of 2.54 (see Table 4).

**Table 4:** Creative performance analysis.

Construct	*M*	SD	Med.	Max	Min
Creative performance	*3.48*	*0.42*	3.54	4.25	2.54

### Path analysis

This study discussed the relationship between creative self-efficacy, three learning engagements (behavioral, affective, cognitive) and creative performance within the framework of engagement theory. The results of the research model validation showed that all six research hypotheses were supported. The details were as followings; the study results showed that creative self-efficacy had a significant positive impact on behavioral engagement (*β* = 0.87, *p* < 0.001); creative self-efficacy had a significant positive impact on emotional engagement (*β* = 0.84, *p* < 0.001); creative self-efficacy had a significant positive impact on cognition engagement (*β* = 0.89, *p* < 0.001); behavioral engagement had a significant positive effect on creative performance (*β* = 0.26, *p* < 0.001); emotional engagement had a significant positive effect on creative performance (*β* = 0.61, *p* < 0.001); and cognitive engagement had a significant positive effect on creative performance (*β* = 0.61, *p* < 0.001).

**Figure 7:**
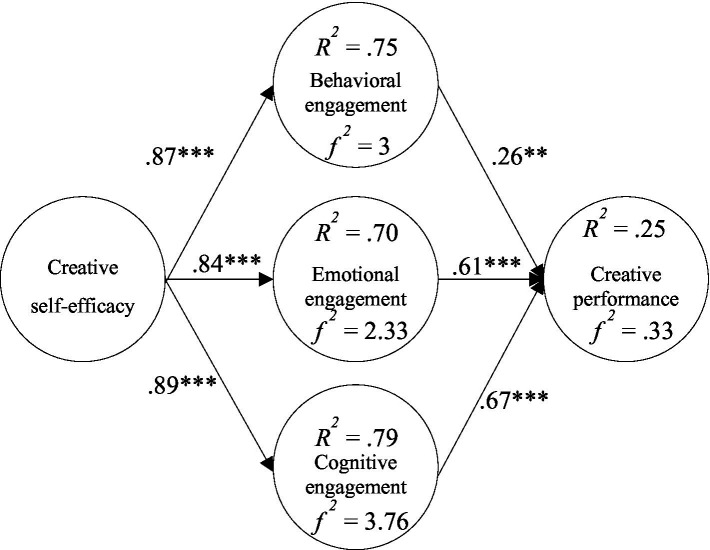
Verification of the research model. ^**^p < 0.01; ^***^p < 0.001.

The explanatory power of creative self-efficacy for behavioral engagement was 75%, and the effect size was 3; the explanatory power for emotional engagement was 70%, and the effect size was 2.33; the explanatory power for cognitive engagement was 79%, and the effect size was 3.76; the explanatory power of the three types of learning engagement on creative performance was 25%, and the effect size was.33 (see Figure 7).

## Discussion

Based on the theory of learning engagement, this study introduced creative self-efficacy and creative performance to construct a research model. According to the assumptions of the model, questionnaires were produced, distributed and recovered. Valid data was obtained through research and analysis, which verified the validity of the model. Further analysis showed the impact of various research variables on the participation of students in higher vocational colleges in art and design courses based on C-STEAM.

C-STEAM emphasizes the integration of disciplines education for cultural inheritance, which provides a new direction for the localization of STEAM education and also innovates the development of excellent local cultural education in China. Among them, the Shangshan culture is a distinctive local culture in the Yangtze River Basin of China. It is the birthplace of the world’s painted pottery civilization, the birthplace of Chinese farming villages and also the world’s rice farming civilization. In the C-STEAM design course, elements such as pottery, stone tool modeling and patterns, village site selection, wooden structure buildings, and rice planting techniques are comprehensively and vividly combined with interdisciplinary concepts, creating a truly diversified, creative and unique learning environment that provides students to learn knowledge and improve their skills, while nourishing their beautiful local inner flows, experiencing the joy of the unity of heart and hand, internalizing culture in their hearts and externalizing them in their actions, and building a more complete knowledge structure for learners.

The “Packaging Design” course integrates the customs, diet, clothing, and artistic features that are unique in local culture and represent people’s daily life. The study results further showed that students had successfully integrated interdisciplinary knowledge into art through the “Packaging Design” course. In the thematic production, in order to solve practical problems, students used local culture as the basis to show the connection between their personal understanding of different disciplines and viewpoints. The creative process was a higher-level understanding of science, and finally built up students’ interdisciplinary knowledge. Jia et al. (2021) also confirmed the evaluation to the comprehensive STEAM approach to academic performance. STEAM education can not only improve students’ ability to conceptualize topics, but also strengthen students’ learning and understanding of interdisciplinary knowledge, and promote students’ overall academic performance in learning activities. The results of this study were consistent with the above research results. C-STEAM curriculum can improve students’ creative self-efficacy, behavioral engagement, emotional engagement, cognitive engagement and creative performance, thereby promote the overall improvement of interdisciplinary knowledge and cultural connotation.

### Creative self-efficacy is positively correlated with learning engagement

The results of this study showed that creative self-efficacy was positively correlated with learning engagement (cognitive, emotional, and behavioral), so H1, H2, and H3 were proven. This is consistent with the similar study by Yang et al. (2022) and Alemayehu and Chen (2021). Creative self-efficacy and learning input helped learners to persevere in learning activities, to strengthen the individual’s belief in task completion, and to enable students to have an inherent and continuous ability to support individual behavior in the production of special projects, that is, learner self-efficacy. The higher the level of self-efficacy, the better the performance of learning cognition, emotion and behavior. In addition, creative self-efficacy and three types of learning engagement have positive effects, but among the three paths, creative self-efficacy has the lowest index of emotional engagement, which Willms (2003) also stated in his research.

### The impact of learning engagement and creative performance is positively correlated

The results of this study represented that learning engagement (cognitive, affective, and behavioral) had a positive correlation with creative performance, so H4, H5, and H6 were proven. Bresó et al. (2011) and Li et al. (2022) also reached the same conclusion in their study. When students gain successful experience through self-investment and demonstrate the skills to perform learning tasks, and then the feedback are satisfied, they are prone to continuously invest in behavior, and more input spent in the learning cycle: the more energy and time spent, the greater the impact on academic feedback. In addition, the three types of learning engagement, cognitive, affective and behavioral, had a positive correlation with creative performance, and among the three significant paths, the path coefficient value of cognitive engagement on creative performance was higher than that of behavioral engagement and emotional engagement. The study resulted was consistent with the study by Marks (2000).

## Conclusion and suggestions

### Conclusion

The purpose of this study was to understand the learning effects of integrating the art and design field in higher vocational colleges based on the concepts of C-STEAM. Based on the learning engagement theory as the research model, six research hypotheses were constructed. The experimental results verified that creative self-efficacy was positively correlated with learning engagement (cognitive, emotional, and behavioral), and learning engagement (cognitive, emotional, and behavioral) had a positive impact on creative performance. In addition, through a 9-week quasi-experimental study of C-STEAM application packaging design, it was found that students in a higher vocational college could skillfully combine interdisciplinary knowledge to create “personalized” packaging works. Therefore, if the concepts of C-STEAM can be popularized in the field of packaging design, it would not only help to integrate all aspects of C-STEAM knowledge and skills into packaging design learning, but would also help to improve the comprehensive design ability and cultural practice ability in other relevant majors. Eventually, the connection between art talent training and market demand can be tied up more quickly.

The importance of STEAM teaching in the field of art and design has been indicated in international research. However, C-STEAM has not been integrated into the curriculum of the current art and design education in China’s higher vocational colleges. There is a lack of relevant specific research in China. Therefore, this quasi-experiment has shown the benefits of C-STEAM for art and design and recommends the promotion of the implementation of this educational method in the field of art and design. For example, during the learning process of the C-STEAM packaging design course, teachers can lead students to apply interdisciplinary knowledge and skills to modern design, accelerate the formation of “cultural-oriented” design concepts in design practice, promote students to build a more complete knowledge structure, and facilitate long-term development. The establishment of the C-STEAM art course includes multi-disciplinary elements, professional elements, and cultural elements as the main body of teaching to approach the goal of the multi-disciplinary focus, inheritance and development, and the cultivation of precision art design professionals in higher vocational colleges. Furthermore, the inheritance and promotion of local traditional culture among young students can be more effectively passed on to future generations. The influence of local culture can be widely spread and social resources can be comprehensively activated.

### Implications

Based on the concept of C-STEAM proposed by Zhan et al. (2022), this study verified the transformation of STEAM education into China’s localization. It was guided by the inheritance of China’s excellent local culture and interdisciplinary knowledge integration education (C-STEAM). With fully considering the laws of creative thinking, the ability to solve practical problems and emotional internalization relied on the most distinctive Chinese local culture – Shangshan culture. Furthermore, to carry out the design teaching experiments, the course maximized the height of interdisciplinary knowledge, integrating with the elements close to students’ daily life in design courses. It not only makes design education full of new contents and more vivid and interesting ideas, but also pulls in the distance between classroom teaching and local life, promotes the activation of classrooms and academic excellence. This study provided a theoretical basis from the perspective the design course, which was the theoretical contribution to the educational research.

In addition, this study constructed a C-STEAM-based design course in Chinese higher vocational colleges from a new perspective, and verified the importance and educational significance of integrating C-STEAM into design courses, by guiding art students in Chinese higher vocational colleges to appreciate, understand, study local culture, and encourage students to apply their multi-disciplinary knowledge of science, technology, engineering, art and mathematics to artistic exploration and creation rich in cultural concepts. Cultivating and developing students’ comprehensive design ability in continuous practice, speeding up the connection between artistic talent training and social needs will achieve win-win results. Effectively promoting the construction of universities and majors with local characteristics accelerates the current development needs of education reform in China. This was also another practical contribution of this study.

### Recommendations

Students’ enthusiasm for learning is the focus of teachers’ concern (Linnenbrink and Pintrich, 2003; Ye et al., 2020). Students’ learning input depends more on professional courses, which are realized through teacher education and teaching (Qian and Wang, 2022). However, related research points out that if teachers in higher vocational colleges only focus on teaching methods, it is not easy to make students feel engaged in learning (Ye et al., 2020). It is more difficult to stimulate creative performance. On the other hand, if teachers insist on “people-oriented,” the more autonomous support is given based on the student-centered approach, and the students’ learning engagement will be higher (Qian and Wang, 2021).

This teaching experiment was found that students’ creative self-efficacy, commitment and creative performance in the study of C-STEAM concept integration of art design courses in higher vocational colleges were all at a high level, but they did not discuss more knowledge, experience and experience of traditional Chinese culture, not the effect of internalization process on learners’ creative self-efficacy and creative performance. Relevant research suggests that traditional cultural elements can be classified into two categories in the course: spiritual cultural elements and aesthetic cultural elements (Qian and Wang, 2022), so that students can absorb the wisdom of the ancients and expand their artistic vision, and be oriented to solve problems in real situations. To narrow the gap between culture, life and art, “learning by doing” and “learning by playing” will help to improve learners’ creative self-efficacy and creative performance.

### Limitations and further study

Limited by the class hours, the lecturing cycle of courses in this study, and the strict adherence to the academic schedule of the college, the number of invited participants was small. Therefore, in future studies, the sample size can be expanded to verify whether the concepts proposed in this study can be more accurate and comprehensive. Meanwhile, the uneven gender ratio in the field of art and design has always been a difficult problem to overcome, mainly due to the fact that most of the learners applying for art and design majors are women (Fan and Ye, 2020). In this study, there was also a large gap in the gender ratio of the participants, with males accounting for 31.11% and females accounting for 68.89%.

Besides, only the current perception of the learners was obtained in this study. In follow-up research, time series analysis methods and observation methods can be used to explore more details of changes in participants’ learning performance in the development of the theme. The interview method can be used to not only understand each learner’s views on this learning method and the degree of acceptance of the course, but also the difficulties encountered by each individual, in order to analyze the factors that affect different students’ participation in C-STEAM-based courses.

## Contribution

This research make three contributions: (1) constructing C-STEAM-based art and design courses in Chinese higher vocational colleges from a new perspective, verifying the importance and educational significance of teaching C-STEAM in art design. The implementation of art design in higher vocational colleges is more conducive to the creation of local characteristic majors and universities. (2) By maximizing the high degree of integration of interdisciplinary knowledge, students are encouraged to apply their multi-disciplinary knowledge of science, technology, engineering, art, and mathematics to artistic exploration and creation that is rich in cultural concepts. The aim is to cultivate and develop students’ comprehensive design ability, and speed up the connection between artistic talent training and social needs to achieve win-win results. (3) With the background of Chinese local culture, this study refined the elements of regional art, and explored the balance between the “imagination” in local culture and the “abstraction” in modern art design, so that students could subtly internalize traditional Chinese culture in order to help maintain the inheritance and development of local culture. In summary, this study provides a theoretical foundation from the perspective of art design for the academic community, and makes further contributions to enriching the diversity of disciplinary elements.

## Data availability statement

The raw data supporting the conclusions of this article will be made available by the authors, without undue reservation.

## Ethics statement

Ethical review and approval was not required for the study on human participants in accordance with the local legislation and institutional requirements. The patients/participants provided their written informed consent to participate in this study.

## Author contributions

All authors listed have made a substantial, direct, and intellectual contribution to the work and approved it for publication.

## Funding

This study was supported by “Research on Shangshan Cultural and Art Value Protection, Activation and Utilization” which was funded by the Humanities and Social Science Youth Foundation of Ministry of Education of China in 2022 (Project No: 22YJC760072).

## Conflict of interest

The authors declare that the research was conducted in the absence of any commercial or financial relationships that could be construed as a potential conflict of interest.

## Publisher’s note

All claims expressed in this article are solely those of the authors and do not necessarily represent those of their affiliated organizations, or those of the publisher, the editors and the reviewers. Any product that may be evaluated in this article, or claim that may be made by its manufacturer, is not guaranteed or endorsed by the publisher.
